# Endogenous plasticity in neuro-rehabilitation following partial spinal cord lesions

**DOI:** 10.3389/fnins.2014.00059

**Published:** 2014-04-07

**Authors:** Bror Alstermark, Lars-Gunnar Pettersson

**Affiliations:** ^1^Section of Physiology, Department of Integrative Medical Biology, Umeå UniversityUmeå, Sweden; ^2^Department of Physiology, Institute of Neuroscience and Physiology, Sahlgrenska Academy at University of GothenburgGothenburg, Sweden

**Keywords:** rehabilitation, partial spinal cord lesion, grasping, corticospinal, rubrospinal, reticulospinal, interneuron, lateral reticular nucleus

## Abstract

Currently, much interest in neuro-rehabilitation is focused on mechanisms related to axonal outgrowth and formation of new circuits although still little is known about the functionality in motor behavior. This is a highly exciting avenue of research and most important to consider when dealing with large lesions. Here, we address endogenous mechanisms with the potential of modifying the function of already existing spinal circuits via associative plasticity. We forward a hypothesis based on experimental findings suggesting that potentiation of synaptic transmission in un-injured pathways can be monitored and adjusted by a Cerebellar loop involving the Reticulospinal, Rubrospinal and Corticospinal tracts and spinal interneurons with projection to motoneurons. This mechanism could be of relevance when lesions are less extensive and the integrity of the neural circuits remains in part. Endogenous plasticity in the spinal cord could be of clinical importance if stimulated in an adequate manner, e.g., by using optimal training protocols.

## Introduction

A major problem following injuries in the CNS is poor recovery of sensorimotor control leaving the subject substantially handicapped for the remaining life. However, there are several remarkable cases of recovery in the literature giving hope (Bach-y-Rita, 1981), but little or no explanation of the underlying mechanisms. Injuries to the spinal cord are often partial and can spare both long and short descending and ascending pathways. Based on experimental findings, we propose a hypothesis for potentiation of transmission in un-injured pathways and which involves spinal interneuronal networks, descending brainstem systems and the Cerebellum.

The spinal cord possesses microcircuits not only for control of reflexes, posture, respiration, locomotion, and scratching, but also for control of voluntary movements like reaching and grasping (cf. reviews and references therein; Baldissera et al., 1981; Alstermark and Lundberg, 1992; Alstermark and Isa, 2012). Behavioral experiments in the cat and monkey showed that reaching and grasping can be controlled via interneuronal circuits in the cervical spinal cord (Alstermark et al., 1981b, 2011; Sasaki et al., 2004). In those experiments, selective spinal cord lesions were made to delineate the different spinal interneuronal systems mediating the command for these movements to forelimb motoneurones and to investigate the control from different descending pathways. It was found that the Corticospinal (CST) and Rubrospinal (RuST) tracts played a major role in the control of reaching and grasping, to the extent that transection of them resulted in complete loss of these movements, whereas the Reticulospinal tract (ReST) did not play any significant role. However, in case of incomplete CST and RuST lesions, there was a fast and significant recovery that could be mediated via the ReST (Alstermark et al., 1987; cf. also Pettersson et al., 2007).

## Background

**Figure 1:**
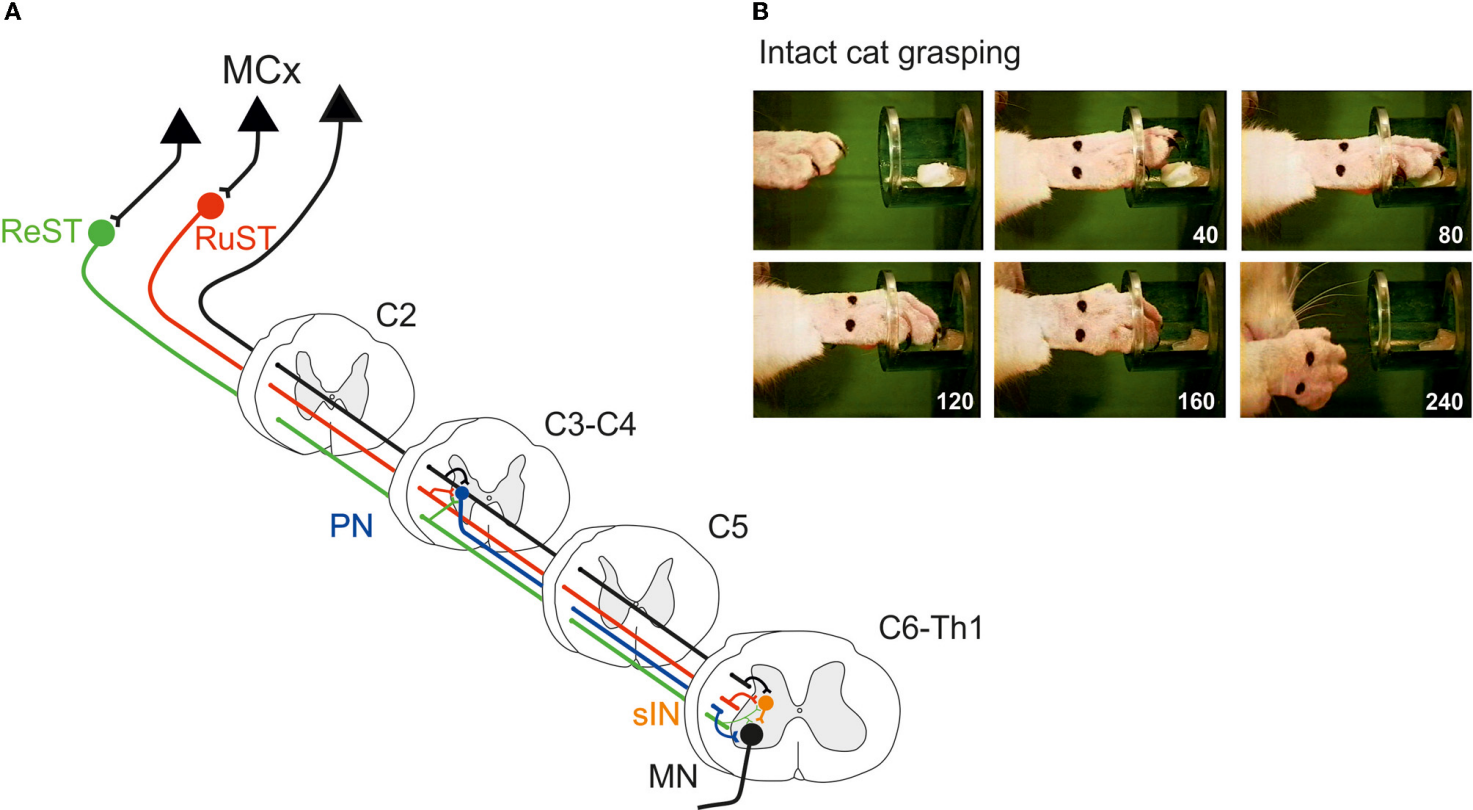
**(A)** Spinal interneuronal systems for control of reaching and grasping movements in the cat. Propriospinal neurons (PNs, blue) in C3-C4 have been shown to mediate the command for reaching to forelimb motoneurones (MN) whereas digit grasping depend on transmission via segmental interneurons (sINs, orange) at the same spinal levels as the forelimb motoneurones (C6-Th1). The motor cortex (MCx) may control these interneuronal pathways via the Corticospinal- (CST, black) tract and, indirectly, via the Rubrospinal (RuST, red) and the Reticulospinal (ReST, green) pathways. **(B)** Example of a digit grasping movement in the intact cat (video, 40 ms resolution; times in milliseconds). To better visualize the components of the movement (claw protrusion and flexion of the digits during grasping followed by flexion at the MCP joint and supination to bring the morsel of food to the mouth) the paw was shaved, the claws painted and the metacarpus marked by two dots. The morsel of food was introduced into the tube via a slidable tray (visible also after removal of the morsel).

Figure 1A illustrates schematically the organization of two spinal interneuronal systems that can mediate the motor commands for reaching, *C3-C4 propriospinal neurons* (PN; labeled in blue) and grasping, *C6-Th1 segmental interneurons* (sINs; labeled in orange). Both systems can be controlled from the cortico- (CST; in black), rubro- (RuST; in red), and reticulospinal (ReST, in green) tracts. The RuST and ReST are controlled from the motor cortex as well as from the deep cerebellar nuclei (not shown). The PNs, sINs, and ReST project directly to the motoneurons (MN). In addition to their motoneuronal projections, the PNs and the sINs project to neurons in the precerebellar Lateral Reticular Nucleus (LRN; not shown). This is illustrated for the sINs in the summarizing Figure 4C.

The preoperative grasping performance is shown for the cat in Figure 1B when retrieving a morsel of food from a tube. Note the combined digit flexion, protrusion of the claws and supination of the wrist after withdrawal from the tube.

**Figure 2:**
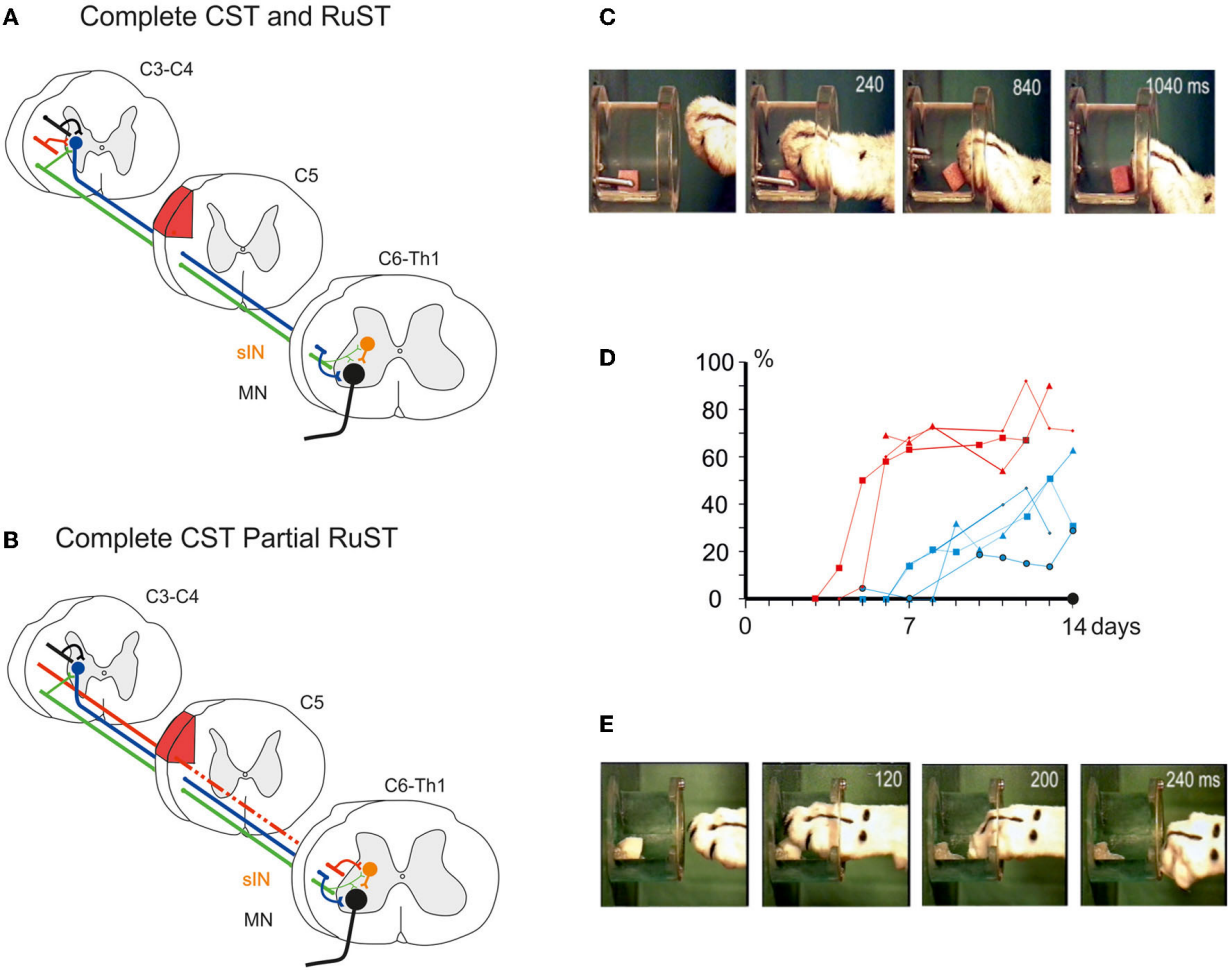
**(A)** Schematic illustration of pathways remaining after a complete lesion (red area) of the DLF in C5/C6 interrupting the CST and the RuST. **(B)**, as in **(A)**, but for an incomplete lesion leaving a small fraction of RuST fibres intact. **(C)**, Loss of digit grasping after the lesion in **(A)** (6 days postoperatively). **(D)** Percentage of trials with successful digit grasping in different experiments during the initial two postoperative weeks after lesion in **(A)** (blue plots; 4 cats) and **(B)** (red plots; 3 cats), respectively. The black dot indicates the absence of successful digit grasping during the first 2 weeks after lesion (A) in case of less frequent training and access to morsels dropped on the floor in unsuccessful trials. **(E)** Successful digit grasping recorded 6 days after the lesion in (**B)**. (**C–E**) Modified from Pettersson et al. (2000).

Figure 2A schematically shows a complete CST and RuST lesion made in the dorsal part of the lateral funiculus (DLF) in C5. Following this lesion, the cat completely lost the digit grasping movements as shown in Figure 2C obtained 7 days postoperatively. The diagram in Figure 2D (blue plots) illustrates the percentage of successful trials in different experiments during the first two postoperative weeks in four different cats with complete CST and RuST lesions. A slow recovery is evident, but the success rate remains lower than 50%. In a similar test, but using less frequent training and allowing taking by the mouth of morsels dropped onto the floor in trials with insufficient grasping, Alstermark et al. (1981b) described an even slower recovery. Only weak toe flexion was observed, beginning from 2–4 weeks and the cats only reached a success rate of about 50% after 6 months. No successful trials were observed within the first 2 weeks (indicated by a black dot in Figure 2D). Presumably, the intensity, time of onset and behavioral paradigm are important for the time course of recovery as emphasized by Sugiyama et al. (2013).

In contrast, if some rubrospinal fibers escaped the lesion as shown in Figure 2B, the recovery was much faster and more complete. Alstermark et al. (1987) showed that if about 50% of the RuST remained intact, as assessed by the amplitude of the descending volley recorded from dissected spinal halves caudal to the lesion, successful digit grasping was observed already in the first experiment 6 days postoperatively. Pettersson et al. (2000) showed that as little as 4% of the RuST was sufficient to markedly improve the recovery. The success rates for three cats with 4–6% of remaining RuST are shown in Figure 2D (red plots). Already within the first week the success rate increased to about 70% of the preoperative value. Performance of digit grasping on the 7th postoperative day is exemplified in Figure 2E. The findings are also summarized in the Supplementary Movie 1.

**Figure 3:**
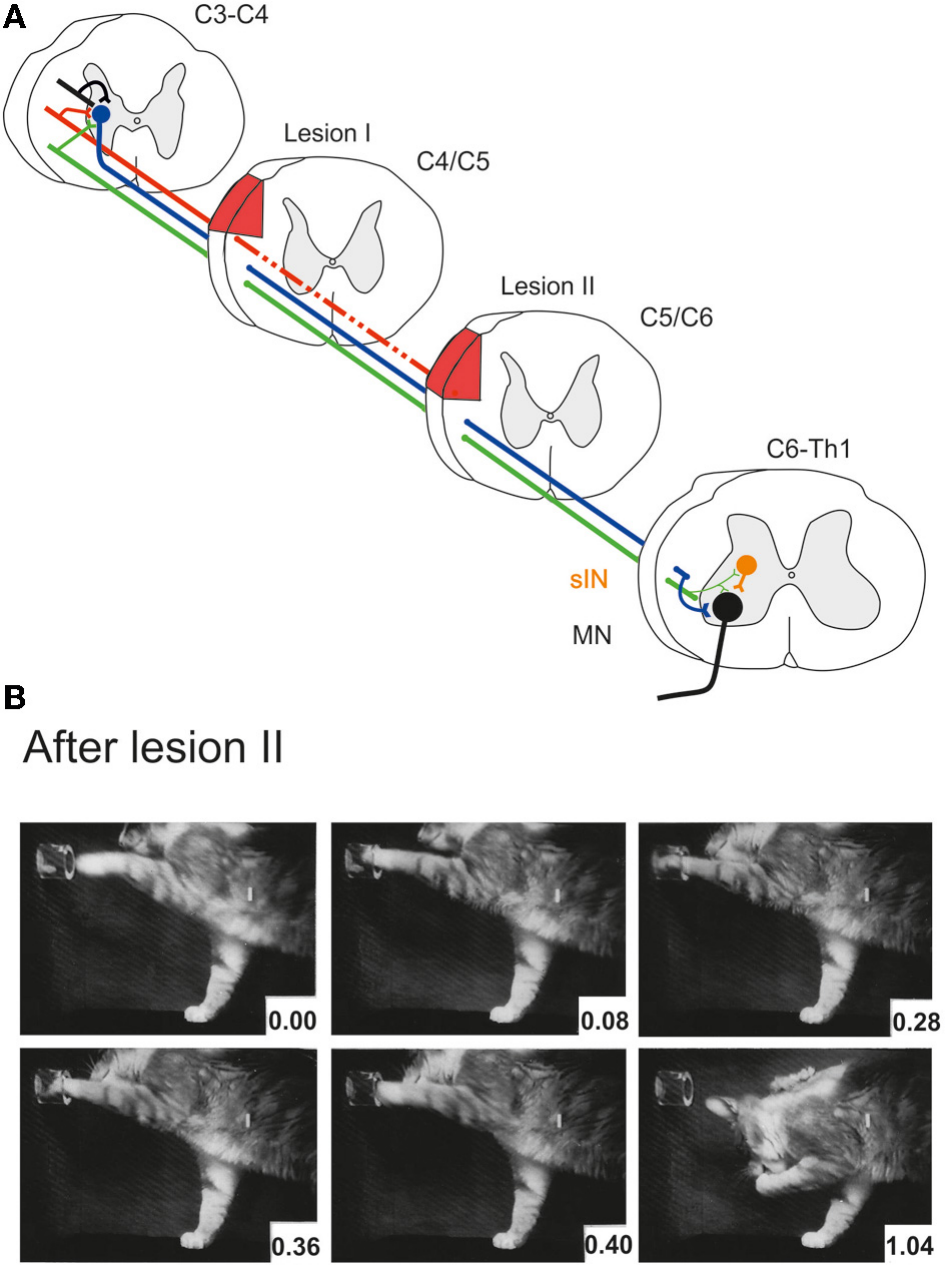
**(A)** Serial lesions of the rubro- and corticospinal tracts in the DLF in two time-separated sessions. Lesion I (complete CST, partial RuST) was made in C4/C5. Lesion II (complete CST and RuST) was made in C5/C6 1 month after lesion I. **(B)** Successful digit grasping in the first experiment (6 days postoperatively) after lesion II. **(B)** Modified from Pettersson (1990).

Alstermark et al. (1987) showed further that 1 month after the incomplete lesion, the remaining RuST fibers were no longer required for performance of the recovered digit grasping movement. If a second lesion of the DLF (denoted lesion II in Figure 3A) was added and which extended more ventrally to transect the remaining intact RuST fibers, digit grasping was present in the first trial 6 days postoperatively. Figure 3A schematically shows that experimental paradigm with two serial lesions of the DLF (lesion I and lesion II) and Figure 3B shows successful retrieval of the morsel of food 6 days after lesion II.

Finally, Alstermark et al. (1987) found that the recovered grasp function could be permanently abolished by adding a third lesion ventrally in C2 transecting the ReST. It was concluded that the intact ReST had taken over the role of the CST and RuST pathways. The RuST fibers remaining after lesion I were suggested to serve as a teacher facilitating the ability to use the ReST to command the movement.

How can such a functional takeover be achieved?

## Hypothesis for functional takeover following partial spinal cord lesion

It is a remarkable finding that as little as 4% remaining RuST fibers, after a DLF lesion, suffice for an improved functional takeover mediated via the ReST. This result strongly argues against that the remaining RuST fibers provided a high degree of specificity in their connections to the sINs. Rather, it suggests that the specificity was provided by the intact ReST and that the RuST fibers only gave a strengthening input to shared sINs.

**Figure 4:**
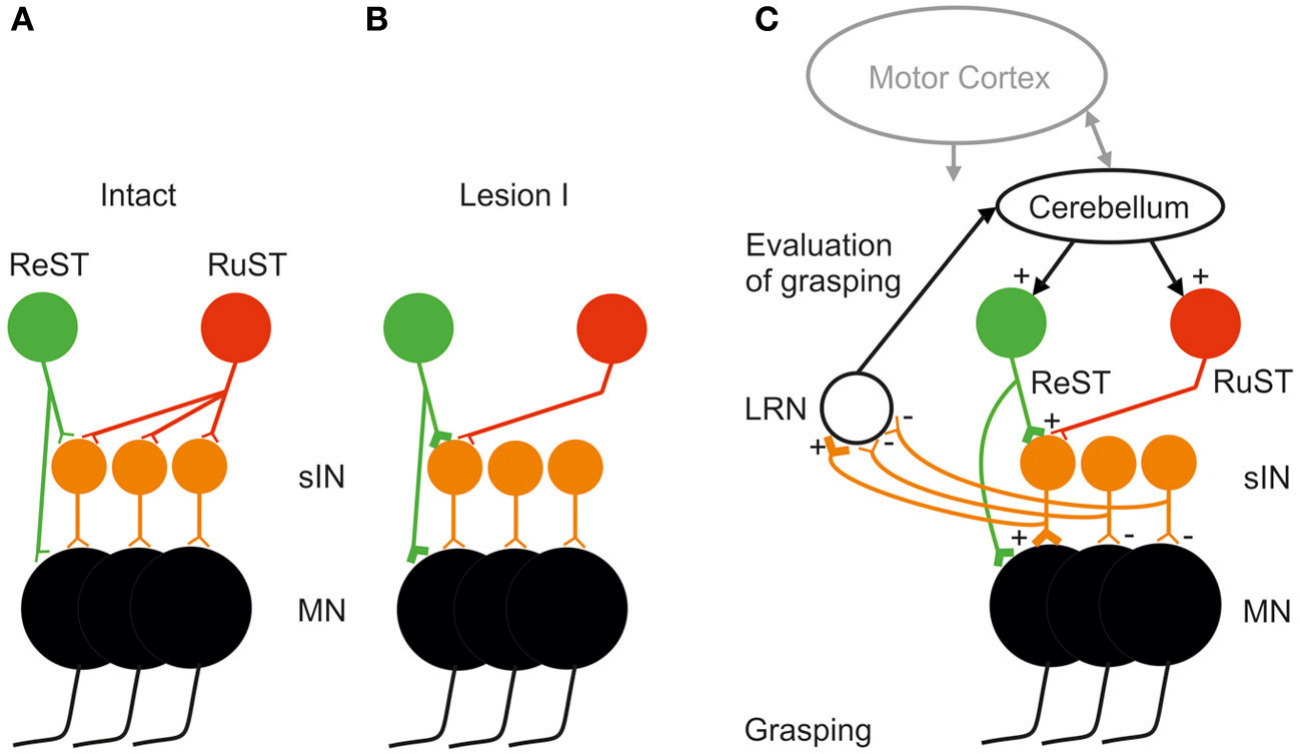
**Tentative explanation of facilitated takeover of digit grasping by the ReST after an incomplete DLF lesion sparing only a small fraction of the RuST (as illustrated in Figures 2B,E). (A)** Before lesion, the illustrated group if sINS used for digit grasping all receive input from the RuST whereas only a minority of them from the ReST. **(B)** After the DLF lesion, the remaining synapses from the RuST (active during attempted digit-grasping) are assumed, by a mechanism of associative plasticity, to facilitate the induction of long-term potentiation in synapses from the ReST onto common sINS. **(C)** Mobilization of the ReST after the DLF lesion as a result of detection of reduced activity in sINS for digit grasping via ascending collaterals from them to the Lateral Reticular Nucleus (LRN). The Cerebellum may increase the activity both of ReST and RuST. The motor cortex is also important in the control of RuST and ReST (see text).

A tentative explanation at network level is illustrated in Figure 4. In A is shown that the intact control of grasping exerted by the RuST is more diversified and involves more sINs than for the ReST. It is assumed that normally there are sINs with convergent inputs from the ReST and RuST. Furthermore, we assume that some of these sINs still receive convergent input after Lesion I. If so, following Lesion I, the only sINs that could mediate the descending command are those shared by the ReST and the remaining RuST. It was proposed that concomitant activity in the remaining synapses of the RuST could facilitate an induction of long term potentiation of synapses of the ReST to common sINs, in a manner of associative synaptic plasticity (Pettersson et al., 2000). During attempted digit grasping the activity of the remaining RuST synapses would then serve to direct synaptic plasticity to ReST synapses terminating on those sINs which are normally used for commanding this movement. (Pettersson et al., 2000, cf. also Pettersson et al., 2007). Since the ReST also has direct connections with the MNs, it is possible that such potentiation could be exerted also on those synapses as indicated in Figure 4B.

In order for a successful takeover, the CNS must evaluate the error in grasping following Lesion I. Such an error evaluation most likely involves the Cerebellum as shown in Figure 4C. A major source of information about spinal interneuronal activity to the cerebellum is provided by mossy fiber input via neurons in the LRN. It has been shown that both PNs in C3-C4 (Alstermark et al., 1981a) and sINs, belonging to the ipsilateral forelimb tract (Ekerot, 1990) send information to the LRN. It has been proposed that the LRN may provide the Cerebellum with an overview of linked motor behaviors like posture, reaching and grasping (Alstermark and Ekerot, 2013). If, following Lesion I, the activity in those sINs controlled by the lesioned RuST decreases, it could be detected by the LRN and Cerebellum. To compensate, the Cerebellum may increase the excitation to the ReST and RuST with convergent projection to sINs. If this compensation leads to more successful grasping, this loop can be further strengthened. The function of the ascending spinal information processed by the LRN may be tested in future experiments by selective inhibition of the LRN neurons using opto-genetic techniques (Fenno et al., 2011; Miesenböck, 2011).

We have intentionally focused on the spinal mossy fiber-cerebellar-bulbospinal loop. There may be a control of errors via the climbing fiber-cerebellar-bulbospinal loop occurring in parallel (Ito, 1984). Also, the interaction between cerebellum-motor cortex- spinal cord is likely to play an important role (Stoodley and Schmahmann, 2010). In fact, it has been shown that there are marked excitability changes occurring in the motor cortex following spinal cord lesions (Isa and Nishimura, 2014). The augmentation of ReST output may therefore also be strengthened by increased activation of cortico-reticular pathways. An important contributor to augmented cortical modulation might be feedback control from visual pathways, as has been shown after complete spinal cord lesions in humans (Hotz-Boendermaker et al., 2011).

## Functional takeover in primates?

**Figure 5:**
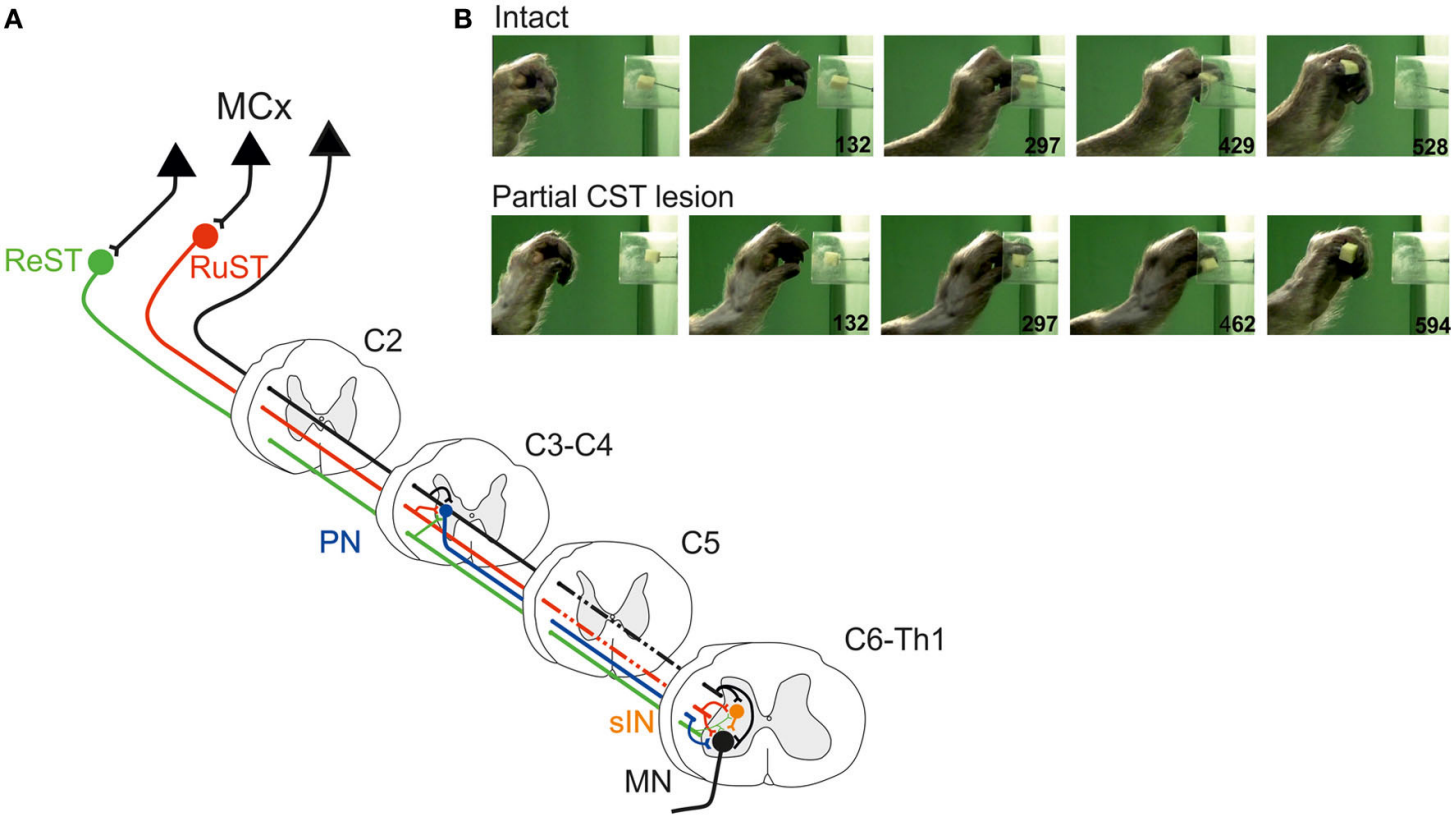
**(A)** As in Figure 1A but for the Macaque monkey illustrating also the direct (monosynaptic) cortico- and rubromotoneuronal pathways (see text for details). **(B)** Precision grip movement (video, time resolution 33 ms) in a macaque monkey before and after an incomplete DLF lesion in C4/C5 with remaining CST (and RuST) fibers to the arm/hand segments.

So far, it has only been possible to study a takeover in function via ReST induced by the RuST in the cat, because of the relatively dorsally located CST (Nyberg-Hansen and Brodal, 1963, 1964) that makes it possible to perform complete CST and partial RuST lesions. In the primate, the axonal locations of the CST and RuST overlap extensively, especially in the ventral part of the lateral funiculus (Poirier and Bouvier, 1966; Bortoff and Strick, 1993) and therefore it is more difficult to make selective lesions. Nevertheless, results from incomplete DLF lesions in the primate may still be of relevance. We show one example in Figure 5 which illustrates the effect of a DLF lesion on precision grip movements using the thumb and the index finger. The schematic circuitry is shown in A. Note the direct motoneuronal connections of the CST (Bernhard and Bohm, 1954), RuST (Holstege et al., 1988), ReST (Riddle et al., 2009), and the C3-C4 PN pathway (Alstermark et al., 1999). The evidence for a disynaptic corticomotoneuronal pathway via the sINs is only indirect, but suggests an effect at least to intrinsic hand muscles (Takei and Seki, 2010, 2013). Figure 5B illustrates precision grip performed the second day after a DLF lesion with incomplete transection in a macaque monkey, sparing 30% of the CST at the level of C4/C5 (Nishimura et al. unpublished data). Note the similarity in pre-and postoperative movements.

A previous report on DLF lesions with complete transection of the CST in C5 (Sasaki et al., 2004) showed successful precision grip during the first day postoperatively but with weaker grip forces so that the fingers slipped more easily and in many trials, more than one attempt were needed for successful grasping. A reduction of preshaping and increased duration of the movement was also observed (Sasaki et al., 2004). The deficits remained during an observation period of 3 months. A comparison of precision grip movements during the second postoperative day after DLF lesions with complete vs. incomplete transection of the CST is shown in the Supplementary Movie 2.

The observation of a swifter precision grip movement after partial DLF lesion in the macaque monkey resembles the findings for the (albeit less dexterous) digit grasping movement in the cat. In all likelihood the precision grip during the early postoperative period depends on the CST- and possibly also on the RuST fibers which escaped the DLF lesion. However, it remains to be investigated if the spared CST and RuST fibers can, in a long-term perspective, induce a take-over of digit grasping by other pathways such as the ReST or via the C3-C4 PN system. It is interesting that a reticulospinal pathway to arm and hand MNs has been demonstrated (Riddle et al., 2009) and that reticulospinal effects are enhanced after pyramidotomy (Zaaimi et al., 2012).

## Using endogenous plasticity to enhance neuro-rehabilitation

The main points presented in this article are:

1. Following a complete CST lesion and partial RuST lesion, the intact cortico-reticulospinal system, ReST, may take over the control of grasping by the help of the remaining RuST fibers. This was proven in the cat, but preliminary findings in the macaque monkey suggest the possibility of a similar mechanism operating in primates.
2. The outcome of the takeover is dependent on the remaining RuST fibers after the partial lesion. The more RuST fibers remaining, the better is the takeover via the ReST. However, a clear effect is observed even with as few as 4% remaining RuST fibers, which hypothetically could be explained by associative synaptic plasticity on common spinal INs.
3. To evaluate the recovery, it is proposed that the Cerebellum may play an important role. It may use information from the last order premotor interneurons by virtue of their projections to the precerebellar LRN. The Cerebellum may then change the activity in the ReST and the surviving RuST neurons to compensate for the loss of control of the lesioned fibers leading to strengthening of excitatory synaptic input from sINs and ReST to MN.

These results and theoretical considerations could have a bearing on clinical rehabilitation in man and may be taken to suggest the following guide lines for the training after partial spinal cord lesions.

1. Start the training as soon as possible after the injury. If the patient cannot perform movements, use mental imaging, visual, tactile and proprioceptive feed-back.
2. Focus the training on skilled movements that the patient normally performs, like handling knife, fork, spoon, and chop sticks when eating, skilled typing using key-board, picking up small items with a few fingers, open/close locks using keys, buttoning a shirt and tying shoe laces.
3. Combine training of postural control when reaching to grasp for an object.

### Conflict of interest statement

The authors declare that the research was conducted in the absence of any commercial or financial relationships that could be construed as a potential conflict of interest.

## Supplementary material

Movie 1 **Movie of two cats showing grasping of a morsel of food from a tube. Cat #1**, the first sequence was recorded preoperatively and the second sequence on the 7th postoperative day after complete transection of the CST and RuST in C5/C6. **Cat #2**, the first sequence was recorded preoperatively and the second sequence on the 7th postoperative day after complete transection of the CST and partial, sparing 4% of the RuST. The third sequence is the same as the second one, but shown at a lower frame rate.

Movie 2 **Movie of two Macaque monkeys showing grasping of a morsel of food from a tube with the thumb and index fingers. Monkey #1**, the first sequence was recorded on the 2nd postoperative day preoperatively and the second sequence after complete transection of the CST C4/C5 (Sasaki et al., 2004). **Monkey #2**, the first sequence was recorded preoperatively and the second sequence on the 7th postoperative day after partial transection of the CST sparing 30% of these fibers (Nishimura et al. unpublished data).
